# Towards guided and automated programming of subthalamic area stimulation in Parkinson’s disease

**DOI:** 10.1093/braincomms/fcac003

**Published:** 2022-01-13

**Authors:** San San Xu, Nicholas C. Sinclair, Kristian J. Bulluss, Thushara Perera, Wee-Lih Lee, Hugh J. McDermott, Wesley Thevathasan

**Affiliations:** Bionics Institute, East Melbourne, VIC, Australia; Medical Bionics Department, The University of Melbourne, East Melbourne, VIC, Australia; Department of Neurology, Austin Hospital, Heidelberg, VIC, Australia; Bionics Institute, East Melbourne, VIC, Australia; Medical Bionics Department, The University of Melbourne, East Melbourne, VIC, Australia; Bionics Institute, East Melbourne, VIC, Australia; Department of Neurosurgery, St Vincent’s Hospital Melbourne, Fitzroy, VIC, Australia; Department of Neurosurgery, Austin Hospital, Heidelberg, VIC, Australia; Department of Surgery, The University of Melbourne, Parkville, VIC, Australia; Bionics Institute, East Melbourne, VIC, Australia; Medical Bionics Department, The University of Melbourne, East Melbourne, VIC, Australia; Bionics Institute, East Melbourne, VIC, Australia; Bionics Institute, East Melbourne, VIC, Australia; Medical Bionics Department, The University of Melbourne, East Melbourne, VIC, Australia; Bionics Institute, East Melbourne, VIC, Australia; Department of Neurology, Austin Hospital, Heidelberg, VIC, Australia; Department of Medicine, The University of Melbourne, Parkville, VIC, Australia; Department of Neurology, The Royal Melbourne Hospital, Parkville, VIC, Australia

**Keywords:** deep brain stimulation, Parkinson’s disease, subthalamic nucleus, evoked potentials, local field potentials

## Abstract

Selecting the ideal contact to apply subthalamic nucleus deep brain stimulation in Parkinson’s disease can be an arduous process, with outcomes highly dependent on clinician expertise. This study aims to assess whether neuronal signals recorded intraoperatively in awake patients, and the anatomical location of contacts, can assist programming. In a cohort of 14 patients with Parkinson’s disease, implanted with subthalamic nucleus deep brain stimulation, the four contacts on each lead in the 28 hemispheres were ranked according to proximity to a nominated ideal anatomical location and power of the following neuronal signals: evoked resonant neural activity, beta oscillations and high-frequency oscillations. We assessed how these rankings predicted, on each lead: (i) the motor benefit from deep brain stimulation applied through each contact and (ii) the ‘ideal’ contact to apply deep brain stimulation. The ranking of contacts according to each factor predicted motor benefit from subthalamic nucleus deep brain stimulation, as follows: evoked resonant neural activity; *r*^2^ = 0.50, Akaike information criterion 1039.9, beta; *r*^2^ = 0.50, Akaike information criterion 1041.6, high-frequency oscillations; *r*^2^ = 0.44, Akaike information criterion 1057.2 and anatomy; *r*^2^ = 0.49, Akaike information criterion 1048.0. Combining evoked resonant neural activity, beta and high-frequency oscillations ranking data yielded the strongest predictive model (*r*^2^ = 0.61, Akaike information criterion 1021.5). The ‘ideal’ contact (yielding maximal benefit) was ranked first according to each factor in the following proportion of hemispheres; evoked resonant neural activity 18/28, beta 17/28, anatomy 16/28, high-frequency oscillations 7/28. Across hemispheres, the maximal available deep brain stimulation benefit did not differ from that yielded by contacts chosen by clinicians for chronic therapy or contacts ranked first according to evoked resonant neural activity. Evoked resonant neural activity, beta oscillations and anatomy similarly predicted how motor benefit from subthalamic nucleus deep brain stimulation varied across contacts on each lead. This could assist programming by providing a probability ranking of contacts akin to a ‘monopolar survey’. However, these factors identified the ‘ideal’ contact in only a proportion of hemispheres. More advanced signal processing and anatomical techniques may be needed for the full automation of contact selection.

## Teaser

Xu *et al*. report that structural anatomy, beta oscillations, evoked resonant neural activity and high-frequency oscillations predict motor response to subthalamic nucleus stimulation in Parkinson’s disease. The predictive model improves with combining neuronal signal information. This work supports the use of objective data to assist programming.

## Graphical Abstract

**Graphical Abstract:**
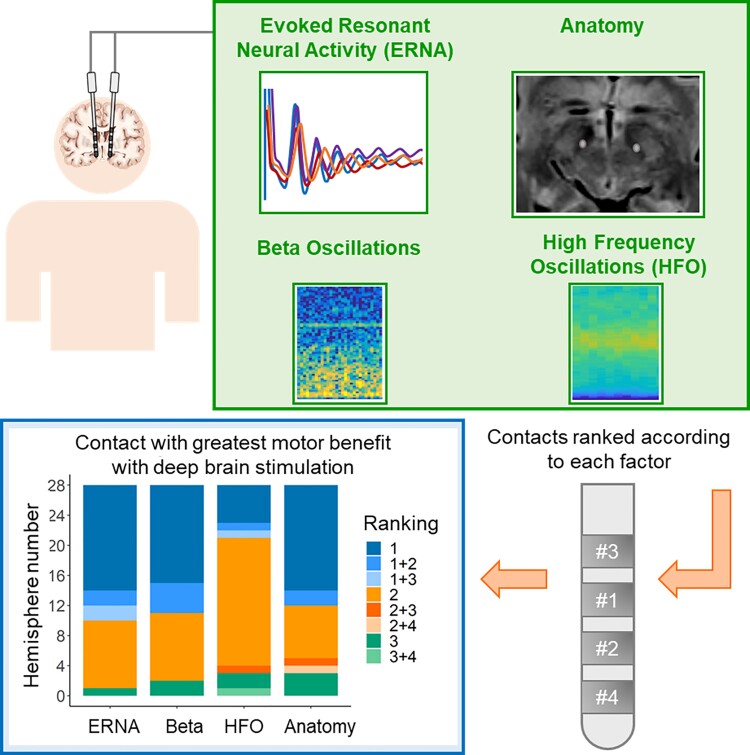

## Introduction

In patients with Parkinson’s disease implanted with subthalamic nucleus (STN) deep brain stimulation (DBS), the most critical step in programming is to select the ideal contact to apply stimulation.^1^ Currently, this is largely achieved by trial and error, often through a ‘monopolar survey’, where DBS is delivered through each contact to provide a ranking according to efficacy and therapeutic window.^1^ Such approaches are time-consuming and rely heavily upon clinician expertise.^2^ Moreover, clinical assessments during programming can be confounded by patient fatigue, ‘stun’ effect and the variable latencies of different therapeutic effects of DBS.^3,4^ Unsurprisingly, many patients gain suboptimal benefit due to inappropriate contact choice.^5,6^ One potential solution is to use objective data, such as anatomical mapping of contacts^7,8^ and neuronal signals recorded from DBS leads during surgery, to guide (and ultimately automate) contact selection. Candidate neuronal signals include beta oscillations,^9–12^ high-frequency oscillations (HFO)^13^ and the recently described evoked resonant neural activity (ERNA).^14,15^ However, the utility of these data, alone or in combination, to aid contact choice for STN-DBS has not been extensively explored.

Thus here, in 28 hemispheres in 14 patients with Parkinson’s disease implanted with STN-DBS, we assessed how well anatomy and neuronal signals recorded in awake patients, can predict, on each lead: (i) the motor benefit from STN-DBS applied through each contact and (ii) the ‘ideal’ contact (yielding maximal motor benefit) to apply DBS. We hypothesized that contact ranking according to all the assessed factors would predict DBS benefit and that a combination of factors would improve this prediction. We hypothesized that the assessed factors would correctly predict the ideal contact in a proportion of hemispheres.

## Materials and methods

### Participants, surgery and clinical programming

We assessed 14 patients (28 hemispheres) with Parkinson’s disease implanted with STN-DBS between February 2016 and February 2018. Fifteen patients were initially recruited but one withdrew before clinical assessment. Informed consent was obtained from all participants. Intraoperative assessments and clinical data have previously been reported in 10 patients.^14^ The study was approved by the St Vincent’s Public (HREC-D 071/14), St Vincent’s Private (R0236-15) and Austin (SSA/15/Austin/266) Human Research Ethics Committees. The patients’ clinical characteristics are summarized in Table 1.^16^

**Table 1:** Characteristics of the 14 study patients including clinical features at the time of initial DBS surgery and chronic DBS parameters recorded at the time of experiments

Patient	Age (years)	Gender	Disease duration (years)	Indication for DBS	Preop LED (mg)	Postop LED (mg)	Hemisphere	Contacts	Current (mA)	Pulse width (μs)	Frequency (Hz)	Postop improvement in UPDRS Part III on/off DBS (%)^a^
1	64	M	8	MF	1250	50	Left	1−/2+	4.2	60	130	59.0
							Right	2−/C+	3.5	60	130	
2	74	F	8	MF	1200	250	Left	2−/1+	4.2	60	130	41.7
							Right	2−/C+	4.2	60	130	
3	62	M	8	MF	1200	250	Left	2−/C+	4.2	60	180	46.2
							Right	2−/C+	4.2	60	180	
4	66	F	10	MF	1250	175	Left	2−/C+	3.5	60	130	61.0
							Right	2−/C+	2.3	60	130	
5	55	M	10	MF	1100	250	Left	2−/C+	2.6	60	130	23.1
							Right	2−/C+	2.7	60	130	
6	56	F	8	MF	600	12.5	Left	2−/C+	3.7	90	180	43.2
							Right	2−/C+	3.5	60	180	
7	63	M	10	MF	1600	350	Left	1−/C+	3.7	90	130	67.4
							Right	2−/C+	4.3	60	130	
8	67	M	8	Tremor MF	800	250	Left	2−/C+	2.7	60	180	25.8
							Right	2−/C+	4.5	90	180	
9	63	F	8	Tremor MF	325	0	Left	2−/C+	2.1	60	180	47.2
							Right	2−/C+	5.1	90	180	
10	54	M	7	MF	100	0	Left	2−/C+	1.8	60	130	59.3
							Right	2−/C+	1.8	60	130	
11	65	F	4	Tremor MF	1050	200	Left	2−/C+	4.5	60	180	85.7
							Right	2−/C+	3.7	60	180	
12	67	M	9	MF	1300	0	Left	2−/C+	4.4	60	180	67.3
							Right	2−/C+	2.7	60	180	
13	64	F	5	MF	575	0	Left	2−/C+	2.3	60	130	68.5
							Right	2−/C+	3.6	60	130	
14	54	M	5	MF	650	0	Left	2−/1+	3.0	60	130	52.8
							Right	2−/1+	2.4	60	130	

DBS, deep brain stimulation; LED, levodopa equivalent dose^16^; UPDRS, Unified Parkinson’s Disease Rating Scale; MF, motor fluctuations.

For contacts: − is cathode, + is anode and C, case. The four contacts on each lead are numbered from ventral to dorsal as follows: 0, 1, 2 and 3.

^a^ The UDPRS was assessed at the time of follow-up which ranged between 3 and 18 months after STN-DBS surgery. Patients were evaluated in the off-medication state.

Quadripolar electrodes (Medtronic, model 3387) were implanted bilaterally targeting the STN during awake neurosurgery as previously described.^14^ The substantia nigra pars reticulata (SNr) was the target for the deepest contact with the middle two contacts targeting the STN and the superior contact targeting the zona incerta.

Postoperatively, DBS programming was performed by two experienced DBS neurologists (W.T., S.S.X.) from a high-volume DBS service (>50 *de novo* implantations annually). Electrodes were localized by manually fusing the preoperative MRI and postoperative CT on a planning station (StealthStation™ S7, Medtronic, Dublin, Ireland). Images were available for visual inspection to assist programming. Clinicians were blinded to the neuronal signal recordings.

At the time of the experimental programming sessions, the mean improvement in Unified Parkinson’s Disease Rating Scale (UPDRS) Part III on DBS (as employed for chronic therapy) compared with the preoperative, off-medication score, was 53.3%, standard deviation 16.5.

### Signal recordings and analysis

Electrode extension leads were externalized intraoperatively and connected to a highly configurable, custom neurostimulator with the ability to deliver tailored stimulation.^17^ Neuronal signals were measured with the patient awake and at rest immediately after lead implantation. Neuronal activity was recorded at each contact in a monopolar configuration and re-referenced to an average of the four unstimulated contacts in the contralateral hemisphere for common mode noise suppression. Local field potentials (LFPs) were recorded during an off-stimulation period of 15 s using a biosignal amplifier (g.USBamp, g.tec medical engineering GmbH, Schiedlberg, Austria) with a 38.4 kHz sampling rate, prior to ERNA assessment. To detect ERNA, burst stimulation was applied to each electrode for 10 s, comprising 10 symmetric 60 µs biphasic pulses in each second, delivered at 3.375 mA and 130 Hz. Recordings were not obtained from one ventral contact (in the SNr) due to a technical fault. After the recording period, the electrode extension leads were connected to the subcutaneous pulse generator under general anaesthesia.

Signals were processed and analysed using MATLAB R2017a (Mathworks, MA, USA). ERNA recordings were zero-phase forward–reverse filtered using a second-order Butterworth high-pass filter (*f*_c_ = 2 Hz) and a second-order Butterworth band-stop filter (*f*_c_ = 49–51 Hz) and further processed using a 21-point centred moving-average filter. Evoked potentials following the last pulse of each burst of stimulation were then extracted and detrended to remove baseline offsets. ERNA was evoked and measurable on at least one contact on every implanted lead. Every implanted lead had at least one contact located within the visible anatomical boundaries of the STN. ERNA power was calculated by squaring the average of the root mean square amplitude from 4 to 20 ms measured at the contact of interest immediately after burst stimulation was applied to each of the other three contacts on the lead.

LFP power was measured over arbitrarily defined frequency bands and detectable in all hemispheres. Peaks in beta and HFO bands were visually identifiable in only a proportion of hemispheres (beta 19/28, HFO 3/28). Beta power was calculated over the 13–30 Hz frequency band.^18^ For beta recordings, Blackman–Harris windowed epochs of 1 s were processed using short-time Fourier transformation. HFO power was calculated over the 200–400 Hz frequency band.^18^ For HFO recordings, sharp spikes, likely from power line interference, were removed using the second-order Butterworth notch filters with cut-off frequencies set to ±0.5 Hz of the spike frequency based on visual inspection. Given the large frequency band occupied by HFOs, epochs of 1 s were processed using Thomson’s multi-taper spectral estimates (20 tapers) to better deliver a smoothed spectrum estimate.^19^

### Contact anatomical localization

Each patient’s preoperative brain MRI was co-registered with the postoperative CT (BRAINSFit, 3D Slicer) and contacts were visually identified from the related artefact. Contacts were ranked in order of proximity to a nominated ideal anatomical location (Supplementary Fig. 1). This ideal location was determined using a local landmark targeting method and, if needed, adjusted after direct visual inspection according to the following method. First, on the native scan of each patient, the adjacent red nucleus was identified and referenced as follows: along the anterior border (‘Bejjani line’), 2 mm inferior to the superior border and 3 mm from the lateral border.^20^ This method is reported to closely approximate the clinically effective stimulation site and to accommodate for interpatient STN variability.^21^ Euclidean distance from each contact to this location was calculated using a dedicated script on MATLAB R2017a, yielding an initial ranking of contacts. Second, each contact was visualized by an expert clinician (S.S.X.) and rankings adjusted if necessary (30/111 contacts), taking into account factors such as the centrality of each contact within the STN and width of the STN at that location (ideally ≥2 mm).^22,23^

### DBS benefit on movement

Motor benefit was assessed 3–18 months after STN-DBS surgery (range: 92–586 days; mean: 306 days) to minimize clinical benefit from ‘stun’ effect and to capture a span of timepoints. The primary motor outcome was the sum of the hemibody motor subscores (Part III, items 20–26) of the UPDRS, recorded by the same movement disorders specialist (S.S.X.). The motor score in all patients was initially recorded ‘off medication/off stimulation’ following the overnight withdrawal of dopaminergic medication and a 45 min DBS wash-out period. Monopolar stimulation was then applied to each contact in both hemispheres simultaneously in a counterbalanced order and motor outcomes were assessed after a wash-in period of 15 min.^3^ The pulse width and frequency were kept constant as per chronic therapy and stimulation amplitude was adjusted if necessary, as follows: reduced by 10% if chronic electrode configuration was bipolar and reduced by 25, 50 or 75% if side effects emerged. Amplitude adjustments due to side effects were accounted for in the statistical model. If the clinician-selected electrode configuration was bipolar or discordant across hemispheres (e.g. top contact in the left and bottom contact in the right hemisphere), the chronic stimulation settings were included as a separate condition in the counterbalanced design. The cathode chosen by the clinician for chronic therapy, in monopolar or bipolar configuration, was classified as the clinician-selected contact. The movement disorders specialist (S.S.X.) and the patient were blinded to the electrode configuration during the ‘on stimulation’ conditions.

### Statistical analysis

To evaluate how well the ‘monopolar survey’ could be predicted, a mixed-effects model (MEM)^24^ evaluated the correlation between the ranking of contacts in each hemisphere according to the predictive factors and the motor benefit in the contralateral hemibody with DBS applied to each contact. Motor benefit was calculated according to the following equation:

$$\begin{array}{rl} & \;\mathrm{UDPRS}\;\mathrm{DBS}\;\mathrm{benefit}\;(\text{\%})\\ & \;=\frac{\mathrm{Off}\;\mathrm{DBS}\;\mathrm{UPDRS}\;-\;\mathrm{On}\;\mathrm{DBS}\;\mathrm{UPDRS}}{\mathrm{Off}\;\mathrm{DBS}\;\mathrm{UPDRS}}\;\times 100 \end{array}$$

In the MEM, patients, and hemispheres (nested into patients), were explored as random effects. Hemisphere was not a significant random effect in the model and removed from the final analysis. The order of conditions and tolerated stimulation level at each contact (25, 50, 75 or 100% of chronic amplitude) were assessed as fixed effects. Only stimulation level correlated with UPDRS DBS benefit [*r*^2^ = 0.39, Akaike information criterion (AIC) 1063.6, *P* < 0.001] and thus retained in the model as a fixed effect. The rankings of contacts according to ERNA power, beta power, HFO power and anatomical location were assigned as fixed effects to build the final MEM.

In assessing how combinations of factors could predict UPDRS DBS benefit, we employed a procedure akin to the ‘best subset regression’ model, where all possible combinations of features were explored in models.^25^ Using the AIC score, we identified the best-fitted model, which required the minimum number of features to achieve the optimal predictive performance (i.e. the lowest AIC score).^26^ This analysis method addresses multicollinearity as the AIC score will penalize a model if the predictive performance does not improve significantly after adding a highly correlated feature.

Spearman’s rank-order correlation was used to evaluate associations between neuronal signals. A repeated-measures ANOVA model compared the DBS benefit in each hemisphere at the first ranked contact according to the factors of interest, the clinician-selected contact and the maximal available DBS benefit. ANOVA was also used to determine: (i) the variation in UPDRS DBS benefit across the four contacts in each hemisphere; (ii) the variation in neuronal signal power across the four contacts in each hemisphere and (iii) the variation in neuronal signal power across the four contacts ranked according to proximity to the nominated ideal anatomical location to apply DBS.

The ANOVA model was built using Minitab 18 (Minitab Inc., PA, USA) and multiple comparisons were corrected using the Tukey method. The MEM was built using the lme4 package^27^ on R Project 4.0.3 (R Core Team, Vienna, Austria). The MEMs were compared using ANOVA with the Bonferroni–Holm method employed for *post hoc* analysis. Corrected *P*-values are shown in all figures. Results were deemed significant if *P* < 0.05.

### Data availability

Anonymized data can be made available for the purpose of replicating procedures and results, subject to an embargo of 24 months from the date of publication.

## Results

### Characterization of neuronal signals and stimulation levels

#### Neuronal signals in the STN region and their relative anatomical location

**Figure 1:**
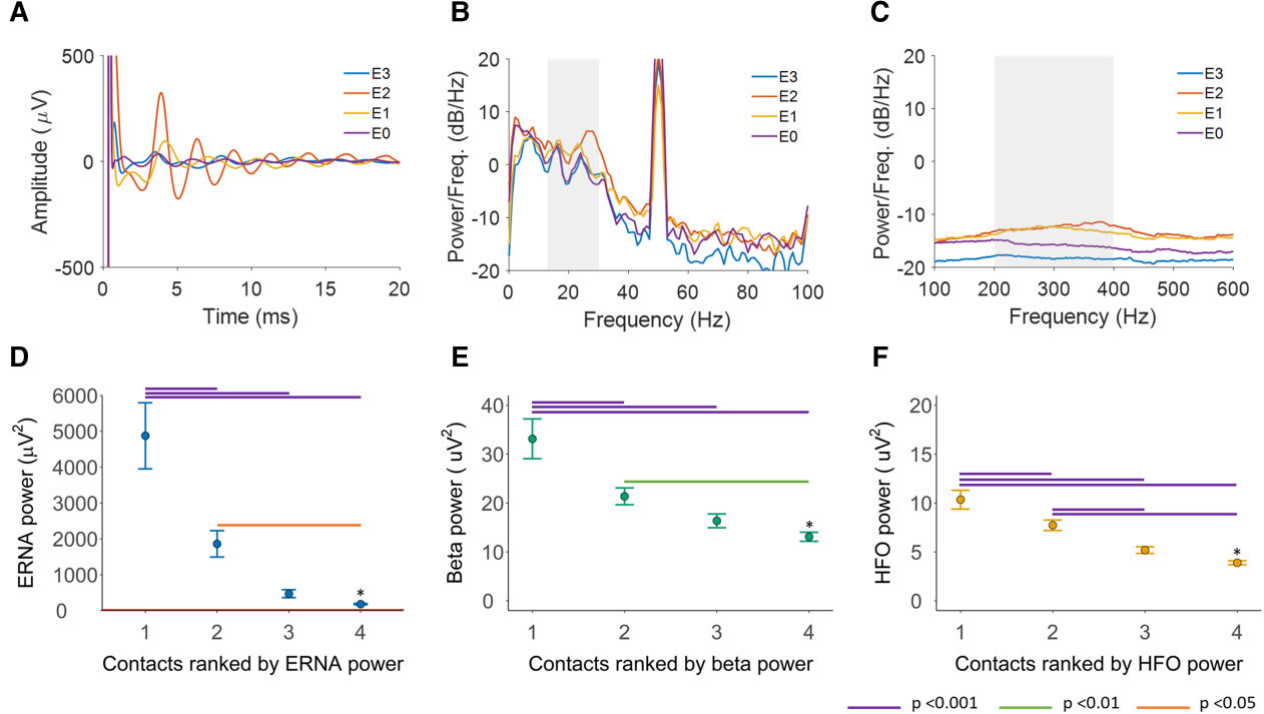
**Neuronal signal characteristics.** (**A**) The ERNA amplitude and power spectral density of (**B**) beta and (**C**) HFO recorded at each of the four contacts in the right hemisphere of participant 1. E0, most ventral contact in the substantia nigra pars reticulata; E1, ventral STN contact; E2, dorsal STN contact; E3, most dorsal contact in the zona incerta. (**D**) ERNA power [repeated-measures ANOVA, *F*(3,94) = 23.4, *P* < 0.001], (**E**) beta power [repeated-measure ANOVA, *F*(3,94) = 22.0, *P* < 0.001] and (**F**) HFO power [repeated-measures ANOVA, *F*(3,94) = 53.1, *P* < 0.001] at contacts ranked according to the neuronal signal power. Red horizontal line in **D** above the *x*-axis represents the power range for beta oscillations and HFO. *At Rankings 1–3, *n* = 28 hemispheres. At Ranking 4, *n* = 27 hemispheres, as recordings were not obtained in one ventral contact due to a technical fault. Bars represent SEMs. ERNA, evoked resonant neural activity; HFO, high-frequency oscillations.

ERNA, beta and HFO power varied across the four contacts in each hemisphere ranked according to signal power [repeated-measures ANOVA, ERNA *F*(3,94) = 23.4, *P* < 0.001; beta *F*(3,94) = 22.0, *P* < 0.001; HFO *F*(3,94) = 53.1, *P* < 0.001, Fig. 1]. Across all contacts, there was a correlation between ERNA power and beta power (*r*_s_ = 0.57, *P* < 0.001), ERNA power and HFO power (*r*_s_ = 0.74, *P* < 0.001) and beta power and HFO power (*r*_s_ = 0.58, *P* < 0.001).

**Figure 2:**
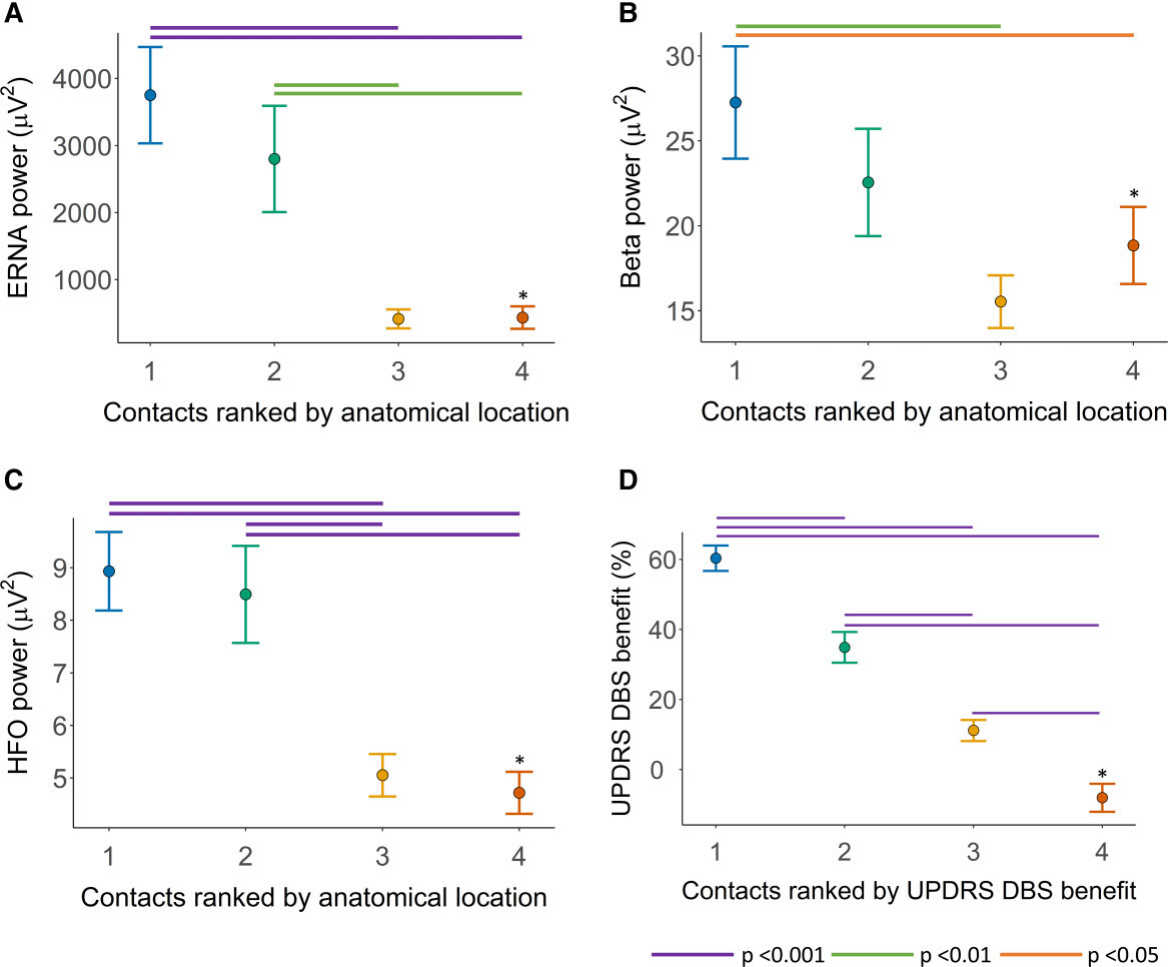
**Anatomical location of neuronal signals and degree of motor benefit with DBS.** (**A**) ERNA power [repeated-measures ANOVA, *F*(3,94) = 11.4, *P* < 0.001], (**B**) beta power [repeated-measures ANOVA, *F*(3,94) = 4.9, *P* = 0.003] and (**C**) HFO power [repeated-measures ANOVA, *F*(3,94) = 19.3, *P* < 0.001] at contacts ranked according to proximity to the nominated ideal anatomical location for DBS in the STN region. (**D**) Hemibody UPDRS DBS benefit at contacts ranked according to the degree of motor benefit with DBS [repeated-measures ANOVA, *F*(3,94) = 137.6, *P* < 0.001]. *At Rankings 1–3, *n* = 28 hemispheres. At Ranking 4, *n* = 27 hemispheres, as recordings were not obtained in one ventral contact due to a technical fault. Bars represent SEMs. ERNA, evoked resonant neural activity; HFO, high-frequency oscillations; DBS, deep brain stimulation; UPDRS, Unified Parkinson’s Disease Rating Scale.

ERNA, beta and HFO power varied across the four contacts ranked according to proximity to the nominated ideal anatomical location [repeated-measures ANOVA, ERNA *F*(3,94) = 11.4, *P* < 0.001; beta *F*(3,94) = 4.9, *P* = 0.003; HFO *F*(3,94) = 19.3, *P* < 0.001]. ERNA, beta and HFO power were greatest at contacts ranked as closer to the nominated ideal anatomical location (Fig. 2A–C).

#### Adjustment of DBS amplitudes due to side effects

Stimulation amplitude was reduced (from chronic therapy) due to side effects (for example, visual change, nausea, capsular side effects) in 29 out of 111 contacts to 75% in 17 contacts, 50% in 11 contacts and 25% in one contact. No amplitude reductions were required at contacts selected by the clinician for chronic therapy. Reductions occurred more often in contacts located further from the nominated ideal anatomical target (10 times in the two best-ranked contacts and 19 times in the two worst-ranked contacts). However, stimulation amplitudes were sometimes reduced at contacts ranked first according to anatomy (five hemispheres), ERNA (two hemispheres), beta (three hemispheres) and HFO (five hemispheres).

### Predicting the ‘monopolar survey’ ranking of contacts according to motor benefit

UPDRS DBS benefit varied across the four contacts in each hemisphere ranked according to efficacy [repeated-measures ANOVA, *F*(3,94) = 137.6, *P* < 0.001, Fig. 2D].

#### Individual predictive factors

**Figure 3:**
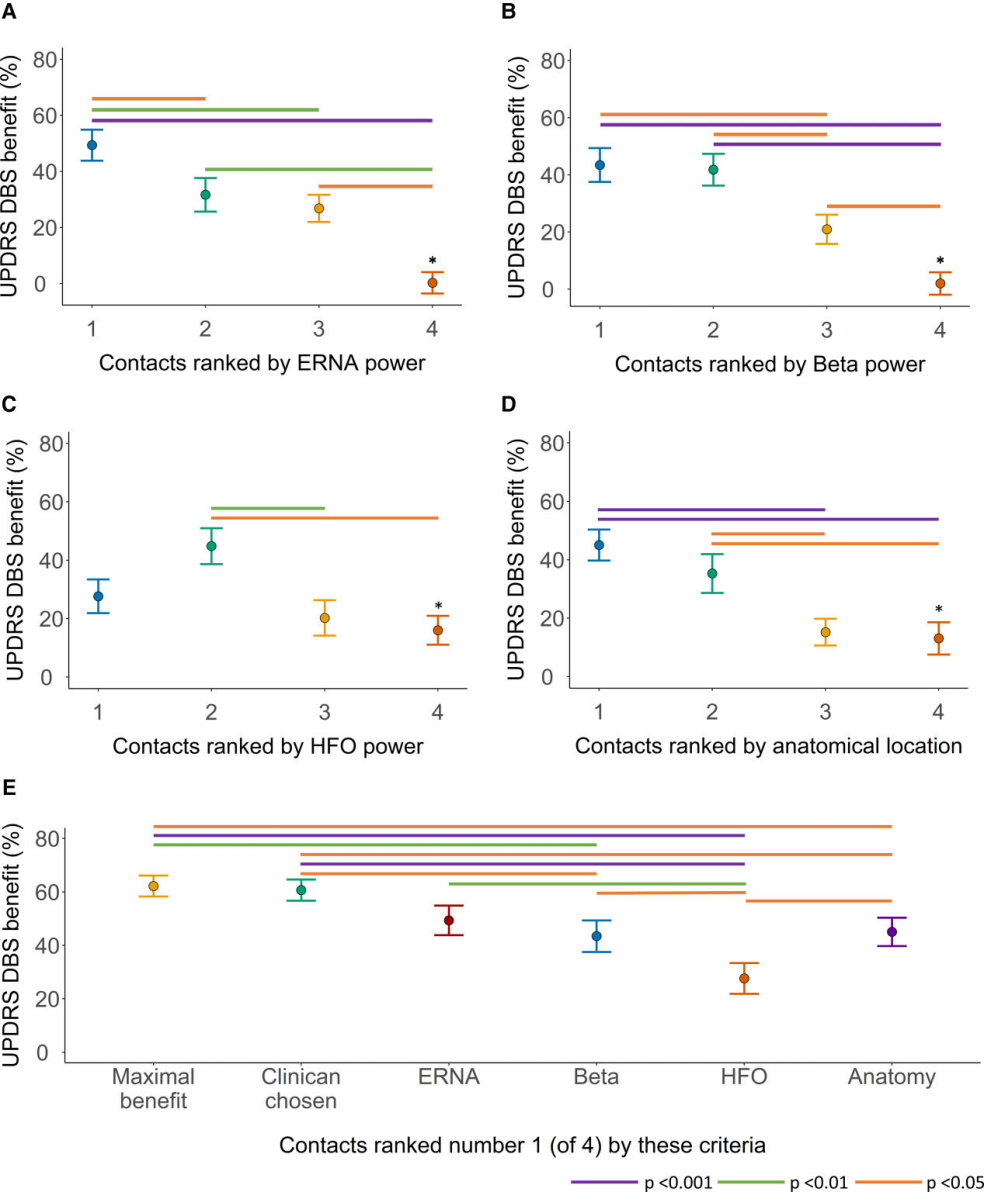
**Relationship between motor benefit with DBS and neuronal signals and anatomical location of contacts.** Hemibody UPDRS DBS benefit at contacts ranked according to (**A**) ERNA power (MEM, *r*^2^ = 0.50, AIC 1039.9, *P* < 0.001), (**B**) beta power (MEM, *r*^2^ = 0.50, AIC 1041.6, *P* < 0.001), (**C**) HFO power (MEM, *r*^2^ = 0.44, 1057.2, *P* < 0.001) and (**D**) proximity to the nominated ideal anatomical location (MEM, *r*^2^ = 0.49, AIC 1048.0, *P* < 0.001). (**E**) Hemibody UPDRS DBS benefit at the contact yielding maximal benefit, at the contact chosen by the clinician for chronic DBS and at the contact ranked first according to ERNA power, beta power, HFO power and proximity to the nominated ideal anatomical location [repeated-measures ANOVA, *F*(5,149) = 11.9, *P* < 0.001]. In **A**–**D**, raw means (dots) and standard errors (bars) are presented in the figures, whilst statistical analyses employed fitted means adjusted for fixed and random effects. *At Rankings 1–3, *n* = 28 hemispheres. At Ranking 4, *n* = 27 hemispheres, as recordings were not obtained in one ventral contact due to a technical fault. AIC, Akaike information criterion; DBS, deep brain stimulation; ERNA, evoked resonant neural activity; HFO, high-frequency oscillations; MEM, mixed-effects model; UPDRS, Unified Parkinson’s Disease Rating Scale.

UPDRS DBS benefit correlated with the ranking of contacts according to ERNA power (*r*^2^ = 0.50, AIC 1039.9, *P* < 0.001), beta power (*r*^2^ = 0.50, AIC 1041.6, *P* < 0.001), HFO power (*r*^2^ = 0.44, 1057.2, *P* < 0.001) and anatomy (*r*^2^ = 0.49, AIC 1048.0, *P* < 0.001) (Fig. 3A–D).

#### Combinations of the predictive factors

The best two-factor predictive model for UPDRS DBS benefit incorporated ERNA power and HFO power rankings (*r*^2^ = 0.57, AIC 1028.0, Table 2). This model was more predictive than the models of either factor alone (ERNA power; *r*^2^ = 0.50, AIC 1039.9, *P* < 0.001 and HFO power; *r*^2^ = 0.44, 1057.2, *P* < 0.001).

**Table 2:** Results of mixed-effects models assessing the degree to which contacts ranked according to ERNA power, beta power, HFO power and anatomical location correlated with UPDRS DBS benefit

Ranked variables in model	Predictive models for UPDRS DBS benefit		
	Conditional *r*^2^	AIC	*P*-value
ERNA	0.50	1039.9	<0.001
Beta	0.50	1041.6	<0.001
HFO	0.44	1057.2	<0.001
Anatomy	0.49	1048.0	<0.001
ERNA and beta	0.53	1037.1	<0.001
ERNA and HFO	0.57	1028.0	<0.001
ERNA and anatomy	0.51	1043.2	<0.001
Beta and HFO	0.52	1040.9	<0.001
Beta and anatomy	0.54	1037.8	<0.001
HFO and anatomy	0.54	1043.1	<0.001
ERNA, HFO and beta	0.61	1021.5	<0.001
ERNA, beta and anatomy	0.54	1039.7	<0.001
ERNA, HFO and anatomy	0.58	1030.6	<0.001
Beta, HFO and anatomy	0.60	1026.5	<0.001
ERNA, HFO, beta and anatomy	0.63	1022.1	<0.001

DBS, deep brain stimulation; UPDRS, Unified Parkinson’s Disease Rating Scale; ERNA, evoked resonant neural activity; HFO, high-frequency oscillations.

The best three-factor predictive model for UPDRS DBS benefit incorporated ERNA power, HFO power and beta power rankings (*r*^2^ = 0.61, AIC 1021.5). This model was more predictive than the two-factor predictive model of ERNA power and HFO power rankings (*P* = 0.006). The inclusion of anatomy ranking into the three-factor model (ERNA power, HFO power and beta power) did not yield a more predictive model (*r*^2^ = 0.63, AIC 1022.1, *P* = 0.15).

### Predicting the ideal contact to apply STN-DBS

Across hemispheres, UPDRS DBS benefit varied between contacts grouped into the following categories: contacts yielding the maximal available benefit, contacts clinically selected for chronic DBS and contacts ranked first according to each predictive factor [repeated-measures ANOVA, *F*(5,149) = 11.9, *P* < 0.001, Fig. 3E].

Within hemispheres, there was a significant difference between the UPDRS DBS benefit at the first ranked contact and the remaining three contacts on each lead according to ERNA power, but not beta power, HFO power or anatomy (Fig. 3A–D, Bonferroni–Holm multiple comparisons).

#### Contacts yielding the maximal UPDRS DBS benefit

The maximal UPDRS DBS benefit in each hemisphere [mean benefit 62.2%, standard error of the mean (SEM) 3.9] did not differ from the UPDRS DBS benefit arising from contacts clinically selected for chronic therapy (mean benefit 60.7% SEM 4.0, Tukey method *P* = 1.0) or contacts ranked first according to ERNA (mean benefit 49.4% SEM 5.5, Tukey method *P* = 0.1). The maximal UPDRS DBS benefit in each hemisphere was greater than the UPDRS DBS benefit arising from contacts ranked first according to anatomy (mean benefit 45.0% SEM 5.3, Tukey method *P* = 0.02), beta (mean benefit 43.4% SEM 5.9, Tukey method *P* = 0.006) and HFOs (mean benefit 27.7% SEM 5.8, Tukey method *P* <  0.001).

However, the UPDRS DBS benefit from contacts ranked first according to ERNA power was not significantly different from contacts ranked first according to beta power or anatomical location. The UPDRS DBS benefit from contacts ranked first according to ERNA, beta and anatomy was greater than contacts ranked first according to HFOs (Tukey method ERNA and HFO *P* = 0.001, beta and HFO *P* = 0.03, anatomy and HFO *P* = 0.01).

**Figure 4:**
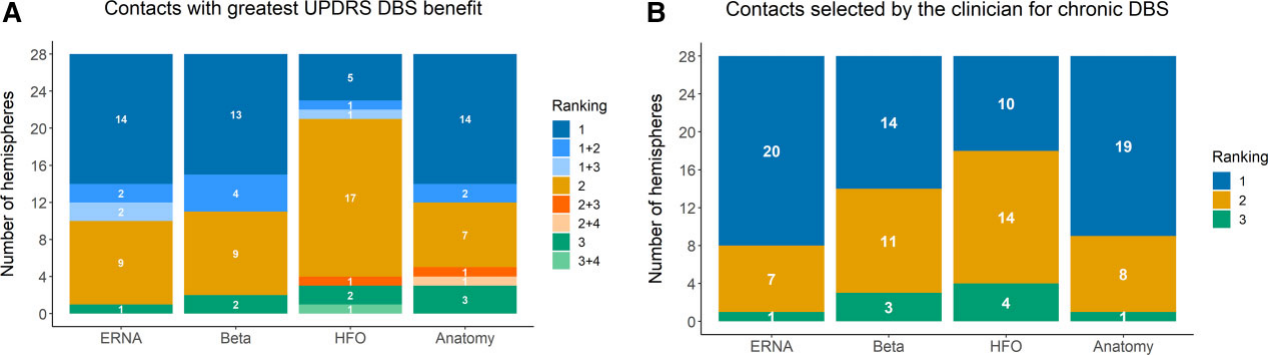
**Relationship between maximal available DBS benefit, chronic DBS location and neuronal signals and anatomical location of contacts**. (**A**) The relationship between contacts yielding greatest motor benefit (UPDRS) with DBS and the ranking of those contacts according to the various factors. In four hemispheres, the UPDRS DBS benefit was the same in two different contacts and both contact rankings are represented. (**B**) The relationship between contacts selected by the clinician for chronic DBS and the ranking of those contacts according to the various factors. DBS, deep brain stimulation; ERNA, evoked resonant neural activity; HFO, high-frequency oscillations; UPDRS, Unified Parkinson’s Disease Rating Scale.

Contacts ranked first according to each predictive factor were the same as those yielding the maximal UPDRS DBS benefit in the following proportion of hemispheres: ERNA 18/28, beta 17/28, anatomy 16/28, HFO 7/28 (Fig. 4A).

#### Contacts clinically selected for chronic DBS

The clinically selected contacts for chronic therapy yielded the maximal UPDRS DBS benefit in 25/28 hemispheres. This contact (cathode for monopolar or bipolar DBS) usually corresponded to the contacts ranked first according to ERNA power (20/28 hemispheres) and anatomy (19/28 hemispheres) and less often to contacts ranked first according to beta power (14/28 hemispheres) and HFO power (10/28 hemispheres) (Fig. 4B).

## Discussion

Here, in patients with Parkinson’s disease implanted with STN-DBS, we assessed how well anatomy and neuronal signals (beta oscillations, HFO and ERNA) recorded intraoperatively in awake patients predicted, on each lead: (i) the ‘monopolar survey’ degree of motor benefit from STN-DBS applied through each contact and (ii) the ideal contact to apply DBS. ERNA, beta oscillations and anatomy were all similarly predictive of UPDRS DBS benefit. The strongest predictive model resulted from combining ERNA, beta and HFO rankings data. The ‘ideal contact’ on each lead was ranked first according to ERNA, beta oscillations and anatomy in a similar proportion of hemispheres and less often according to HFOs. Contacts ranked first according to ERNA (but not the other factors) yielded significantly greater UPDRS DBS benefit than the remaining three contacts on each lead.

Several limitations of this study need to be acknowledged. We evaluated how the assessed factors could predict the efficacy of STN-DBS across contacts on each lead. Randomized controlled trials usually determine such efficacy according to quality of life and disability.^28–30^ Conversely, our single-session experimental study could only assess acute motor effects of DBS and was thus prone to confounds such as stimulation carry-over effects and patient fatigue.^3,31^ However, order effects were non-significant in our statistical analyses. Moreover, the long-term clinical relevance of our results is supported by the high concordance between the clinician-selected DBS contact and the contact identified as ideal during the study. Our sample size may have been insufficient to detect significant differences across all comparisons—but was powered to discriminate clinically relevant outcomes at an individual level, consistent with previous studies of a similar nature.^9,10,12^ We employed DBS parameters that were used for chronic therapy, only adjusting amplitudes in response to side effects. This may have favoured outcomes from the contacts clinically chosen for chronic DBS with stimulation levels being excessive or insufficient at other locations. Excessive DBS can degrade motor performance, but the clinical impact of this is considered modest.^32^ In contacts further from the neural target, increasing DBS can produce greater motor benefit,^33^ though a saturation of benefit can be observed.^34^ It should be noted that the usual treating clinicians did assess the anatomical location of electrodes to help guide programming. This may have increased the concordance between anatomy and the contacts clinically selected for chronic DBS. The duration of experiments was limited by patient tolerance. Thus, we applied DBS bilaterally and performed a comprehensive motor evaluation on each hemibody. Our results could be confounded by ipsilateral effects of DBS but these confer only around a 20% motor benefit in Parkinson’s disease.^35,36^

Here, we provide evidence that contact selection could be guided using objective data. All the assessed factors could (variably) predict the degree of motor benefit with DBS applied through the contacts on each lead—akin to the results of a ‘monopolar survey’. Such information could help narrow programming choices to minimize time costs and error rates associated with DBS programming. Inadequate programming is a common cause of DBS failure^5^ and expert reprogramming improves clinical outcomes.^6^ Thus, using objective data to guide programming could improve reliability and expedite identification of the ideal DBS location, reducing the treatment burden.

Supporting long-term relevance, neuronal signals recorded during electrode implantation predicted clinical outcomes many months after surgery. This is significant, as whilst frequent DBS adjustments are often employed early after implantation due to varying ‘stun’ effect, of greatest importance is optimally applied long-term DBS.^37^ The most vital aspect of contact selection is to identify the single best contact or best combination of contacts to apply chronic DBS. The ideal contact was almost always ranked as first or second according to ERNA, beta oscillations and anatomy. However, these factors ranked the ideal contact as first in only around two-thirds of hemispheres or less.

Combining information from the assessed factors did improve prediction of the degree of motor benefit with STN-DBS at a group level. Thus, each factor may only account for a proportion of the information required to determine the ideal stimulation location. However, the model combining all neuronal signal data did not improve by adding anatomy data, suggesting some redundancy in information. Each factor is, by virtue of their intrinsic properties or the analysis method employed, associated with the dorsal STN region. There may well be better methods of capturing and analysing each of the assessed factors to improve their individual performance in localizing the ideal STN-DBS location. For example, a potential confound here could be the 3 mm spacing between the midpoint of adjacent contacts. The ideal DBS location may be situated between these contacts. Greater spatial resolution, afforded by leads with smaller between-contact spacing and/or directional arrays, could improve the predictive performance of all the factors assessed.

ERNA has many attributes suggesting it could be an ideal neuronal biomarker to tailor STN-DBS. For example, unlike spontaneously occurring LFPs, ERNA occurs with a predictable latency after DBS pulses and is of much larger amplitude. ERNA has a complex waveform with many measurable features such as amplitude, frequency and decay function. In this study, for the purpose of localizing DBS in the STN region, we elected to assess the root mean square amplitude of ERNA over a specific time window and evoking stimulus. However, it is possible that other ERNA variables may better suit this purpose, which will be the focus of future work.

Unlike such evoked activity, there is already a large body of work exploring the relationship between spontaneous LFPs recorded from the STN, especially beta oscillations and motor deficits of Parkinson’s disease.^38–41^ For example, the utility of beta power to guide STN-DBS programming on directional leads has been specifically assessed, with similar findings to this study (the ideal contact was correctly identified in 63% of leads).^10^ It is possible that metrics of beta oscillations other than power may be more predictive of the ideal STN-DBS location, such as spectral divisions,^42–45^ beta burst duration^46^ and the spatial distribution of oscillations.^18,47,48^

Compared with beta-band activity, the relationship between HFOs and Parkinson’s disease motor function remains poorly understood.^44,49,50^ Intriguingly, we found that the contacts ranked second according to HFO power produced the greatest UPDRS DBS benefit. One explanation is that HFOs recorded in the STN could play a prokinetic role, being enhanced with voluntary movement and dopaminergic medication.^50,51^ Indeed, a negative correlation between HFO power and akinesia and rigidity is reported in some studies.^49,50^ However, simply assessing HFO power may not accurately represent the complexity of activity over the broad 200–400 Hz band. For example, shifts in spectral divisions of HFO occur after levodopa administration and correlate with motor scores.^13^ Phase–amplitude coupling of HFOs with beta oscillations has been measured at clinically effective contacts^52^ and is attenuated by dopamine administration and DBS.^13,44,50^

A significant issue that may confound the recording of neuronal signals from recently implanted DBS electrodes is the microlesion or ‘stun’ effect. For example, stun effect may reduce or abolish beta activity soon after electrode insertion.^53^ In this study, beta and HFO peaks were identified in only 68% and 11% of hemispheres, respectively. We, therefore, measured LFPs by simply calculating power over arbitrarily defined frequency bands (e.g. 13–30 Hz for beta). ERNA, being an evoked signal and much larger than LFPs,^14,19^ may not be as prone to microlesioning effects (although this remains to be formally assessed). Indeed, we observed the decaying oscillatory waveform of ERNA in every lead, in all patients in this cohort. Regardless, ERNA power did not perform better than beta power when directly comparing their performance in the single factor models to predict the degree of motor benefit with DBS. However, ERNA more often predicted the contacts clinically selected for chronic DBS. The relative performance of these different signals to guide programming when they are recorded without any microlesion effect (e.g. from implanted sensing devices) is unknown.

Another potential confound of neuronal signal recordings from DBS electrodes during surgery is the level of anaesthesia. In this study, recordings occurred in awake patients. Crucially, LFP measurements are typically greatly diminished or abolished by general anaesthesia.^54,55^ This is a significant limitation in the clinical application of intraoperatively acquired LFPs to guide clinical management, given the global trend towards implanting DBS under general anaesthesia.^56^ In contrast, the distribution and magnitude of ERNA are relatively preserved under deep general anaesthesia with volatile agents or propofol.^55^ The ability of ERNA recorded under general anaesthetic to predict the ideal location for STN-DBS will be assessed in future work.

Clinician aids have already been developed using the anatomical mapping of contacts to assist DBS programming.^7,8,57^ Importantly, our study highlights that, like the other assessed factors, structural anatomy imperfectly identified the ideal STN-DBS contact. Interestingly, the addition of anatomical information to neuronal signal rankings did not improve the predictive model. As ERNA,^14^ beta oscillations^58–61^ and HFO^18,49^ all tended to localize to the dorsal STN, perhaps this rendered structural anatomy redundant in identifying this location. However, when considered alone, anatomy was similarly predictive of the ideal contact to apply STN-DBS as ERNA and beta. Advanced imaging techniques such as tractography and modelling volumes of tissue activated could improve the performance of anatomy for this function. Moreover, such methods could complement the functional information provided by neuronal signals, for example by identifying pathways relevant to side effects.^62–64^

The insights revealed by this study are important to understand, given the established use of anatomy to localize STN-DBS and the emerging availability of commercial intraoperative recording systems^65,66^ and implantable pulse generators capable of monitoring bioelectric signals.^67^ Our results also highlight the potential utility of ERNA, a novel neuronal signal with attributes that have barely been explored. In this study, we analysed ERNA that was recorded intraoperatively. It remains to be seen whether ERNA can be recorded from implantable pulse generators (as described for beta oscillations). Such chronic ERNA recordings would require a high sampling rate (>1000 Hz) and a sufficient window to observe ERNA’s decaying oscillation morphology, for example, by skipping occasional pulses in otherwise continuous trains of therapeutic DBS. We found that ERNA performed as well or better than anatomy and beta oscillations in predicting the ideal contact to apply STN-DBS. However, adding LFP information to ERNA data improved this prediction. Such combining of data increases the signal recording and processing demands and the added complexity may impact reliability. Future studies could assess whether there are better methods to analyse each factor to improve their individual performance in predicting the ideal location to apply STN-DBS.

## Supplementary Material

fcac003_Supplementary_Data

## Abbreviations

AIC =: Akaike information criterion
DBS =: deep brain stimulation
ERNA =: evoked resonant neural activity
HFO =: high-frequency oscillations
LED =: levodopa equivalent dose
LFP =: local field potential
MEM =: mixed-effects model
MF =: motor fluctuations
SNr =: substantia nigra pars reticulata
SEM =: standard error of the mean
STN =: subthalamic nucleus
UPDRS =: Unified Parkinson’s Disease Rating Scale
