# Supplementary material for: Towards guided and automated programming of subthalamic area stimulation in Parkinson’s disease
Source: Brain Commun. 2022 Jan 13;4(1):fcac003. doi: 10.1093/braincomms/fcac003 (PMC8833293; doi:10.1093/braincomms/fcac003)

**Supplementary figure 1: Representative images from a patient of the nominated ideal anatomical location to apply deep brain stimulation.** Fluid-attenuated inversion recovery MRI scan on axial views (A) and coronal views (B). In the same patient, a schematic diagram of the location of the quadripolar electrode array relative to the nominated ideal anatomical location to apply DBS (C). LSTN = left STN. RSTN = right STN. Green dot = nominated ideal anatomical location. Red dotted line = Euclidean distance from the E1 contact to the nominated ideal anatomical location. A = anterior. R = right. S = superior. The four contacts on each lead are numbered from ventral to dorsal as follows; E0, E1, E2, E3.


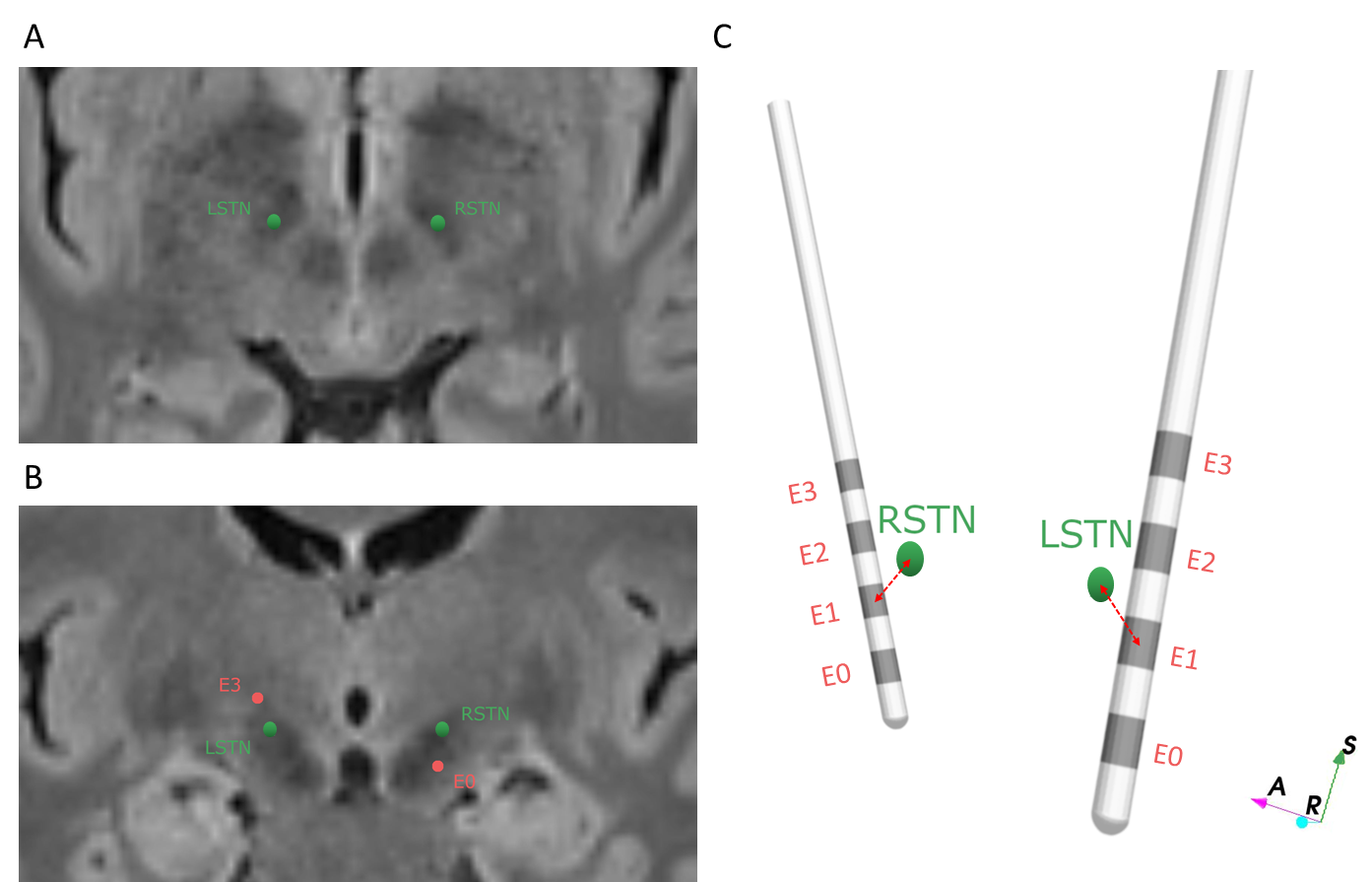

Supplement: fcac003_Supplementary_Data [file fcac003_supplementary_data.docx]
